# Creation of a new class of radiosensitizers for glioblastoma based on the mibefradil pharmacophore

**DOI:** 10.18632/oncotarget.27933

**Published:** 2021-04-27

**Authors:** Sateja Paradkar, James Herrington, Adam Hendricson, Piyasena Hewawasam, Mark Plummer, Denton Hoyer, Ranjini K. Sundaram, Yulia V. Surovtseva, Ranjit S. Bindra

**Affiliations:** ^1^Department of Therapeutic Radiology, Yale University School of Medicine, New Haven, CT 06510-8034, USA; ^2^Yale Center for Molecular Discovery, Yale University, West Haven, CT 06516-0972, USA; ^3^Department of Pathology, Yale University School of Medicine, New Haven, CT 06510-8034, USA; ^*^These authors jointly directed this work

**Keywords:** glioblastoma, radiosensitizers, mibefradil, DNA repair, alternative non-homologous end joining

## Abstract

Glioblastoma (GBM) is the most common primary malignant tumor of the central nervous system with a dismal prognosis. Locoregional failure is common despite high doses of radiation therapy, which has prompted great interest in developing novel strategies to radiosensitize these cancers. Our group previously identified a calcium channel blocker (CCB), mibefradil, as a potential GBM radiosensitizer. We discovered that mibefradil selectively inhibits a key DNA repair pathway, alternative non-homologous end joining. We then initiated a phase I clinical trial that revealed promising initial efficacy of mibefradil, but further development was hampered by dose-limiting toxicities, including CCB-related cardiotoxicity, off-target hERG channel and cytochrome P450 enzymes (CYPs) interactions. Here, we show that mibefradil inhibits DNA repair independent of its CCB activity, and report a series of mibefradil analogues which lack CCB activity and demonstrate reduced hERG and CYP activity while retaining potency as DNA repair inhibitors. We present *in vivo* pharmacokinetic studies of the top analogues with evidence of brain penetration. We also report a targeted siRNA-based screen which suggests a possible role for mTOR and Akt in DNA repair inhibition by this class of drugs. Taken together, these data reveal a new class of mibefradil-based DNA repair inhibitors which can be further advanced into pre-clinical testing and eventually clinical trials, as potential GBM radiosensitizers.

## INTRODUCTION

Glioblastoma (GBM) is the most common primary malignant tumor of the central nervous system (CNS). The current standard of care involves maximal surgical resection followed by radiation therapy (RT) with adjuvant temozolomide (TMZ) chemotherapy. Despite high doses of RT and TMZ, most patients have a recurrence within 1–2 years, and the overall prognosis is dismal [1, 2]. As most recurrences are locoregional in GBM, there is great interest in testing novel strategies to radiosensitize these tumors as a means to improve disease control [3].

DNA repair inhibitors have shown great promise as tumor cell radiosensitizers [4, 5]. Cells utilize several DNA double-strand break (DSB) repair pathways to repair DNA damage induced by irradiation (IR). The two major pathways involved in the repair of DSBs are non-homologous end joining (NHEJ) and homologous recombination (HR) [6]. NHEJ is the predominant repair pathway active during the G0/G1 and G2/M phases of the cell cycle, while HR is active during the S phase of cell cycle during which a sister chromatid becomes available as a homology template. NHEJ is further sub-divided into two pathways, canonical NHEJ (cNHEJ) and non-canonical NHEJ. The latter has been given multiple names, and likely consists of several alternative pathways, with alternative NHEJ (alt-NHEJ) being used frequently [7]. Alt-NHEJ requires end resection of the DSB, followed by single strand annealing using microhomologies. This pathway repairs only 0.5–1% of total DSBs, but serves as a crucial back-up pathway for both NHEJ and HR and for the repair of complex DNA lesions arising from IR-induced damage [8].

We previously published a microplate-based assay to measure both HR and alt-NHEJ simultaneously in live cells, termed EJ-DR [9]. The EJ-DR assay was utilized in a high-throughput chemical screen for novel DNA repair inhibitors, which identified the T-type and L-type calcium channel blocker (CCB), mibefradil, as a selective inhibitor of alt-NHEJ repair. We demonstrated that mibefradil could radiosensitize GBM cells *in vitro* [11], which was also shown by another group using orthotopic, *in vivo* GBM models [10, 11]. A Phase I dose-escalation study was then initiated by our group in a cohort of recurrent GBM patients to determine the maximum tolerated dose of mibefradil in combination with hypofractionated RT (NCT02202993). While several intriguing responses were observed, including one complete radiographic response, significant dose-limiting toxicities were reported [12]. These included sinus bradycardia (28%) and QT interval elongation (28%). The drug-induced QT interval prolongation was largely due to the potent inhibition of the human Ether-à-go-go-Related Gene (hERG) potassium channel by mibefradil, which is known to increase the risk for cardiac arrhythmias. Moreover, mibefradil was previously FDA-approved for hypertension, but then withdrawn owing to drug-drug interactions due to the potent inhibition of the cytochrome P450 enzymes (CYPs) involved in drug metabolism [13, 14].

Based on these findings, we sought to create a new class of radiosensitizers which retained mibefradil’s activity as a DNA repair inhibitor, but showed reduced hERG and CYP450 enzyme inhibition. We found that the CCB ability of mibefradil is non-essential for its ability to inhibit alt-NHEJ. Through structure activity relationship (SAR) analysis, we created and synthesized a series of 140 analogues and profiled them using EJ-DR assays. These compounds were found to be potent alt-NHEJ inhibitors, with reduced CCB activity, as well as attenuated hERG and CYP450 inhibition. We then tested the pharmacokinetic parameters of the synthesized analogues and validated their ability to cross the blood-brain barrier (BBB) and accumulate in mouse brain tissue, at levels similar to that observed with mibefradil. Finally, through the knockdown of DNA damage response (DDR) proteins in the high-throughput imaging-based assay, we identified potential targets or regulators of mibefradil, which phenocopied the selective inhibition of alt-NHEJ over HR. Overall, this study provides a framework for the development of superior mibefradil analogues, with reduced off-target effects and improved potency. These analogues could further be tested as radiosensitizers in *in vivo* models and eventually clinical trials for improving RT efficacy in GBMs.

## RESULTS

### Development of a 384-well high content imaging EJ-DR assay

To support the rapid screening of mibefradil analogues, we adapted the previously published EJ-DR assay from our group to a 384-well imaging-based assay [9]. The EJ-DR system was made in the U2OS cell line, which provides a simultaneous read-out of alt-NHEJ and HR activity in cells in response to induced DSBs. The cell line consists of a repair template and a reporter vector. When both these elements are chromosomally integrated, cleavage of a restriction enzyme site in the repair template, induced by ligand addition, causes a DSB. Depending on which DSB repair pathway is utilized, cells either express GFP if HR is used to repair the break site using the downstream homology template near the restriction enzyme site, or RFP if alt-NHEJ is involved in ligation of the cut ends. In previous publications, we have referred to this type of repair as mutagenic, or non-canonical NHEJ. Subsequent studies from our group and others have confirmed that the EJ-DR assay provides a robust readout of alt-NHEJ, and thus we now use this term to describe the repair pathway being monitored [15–17].

**Figure 1:**
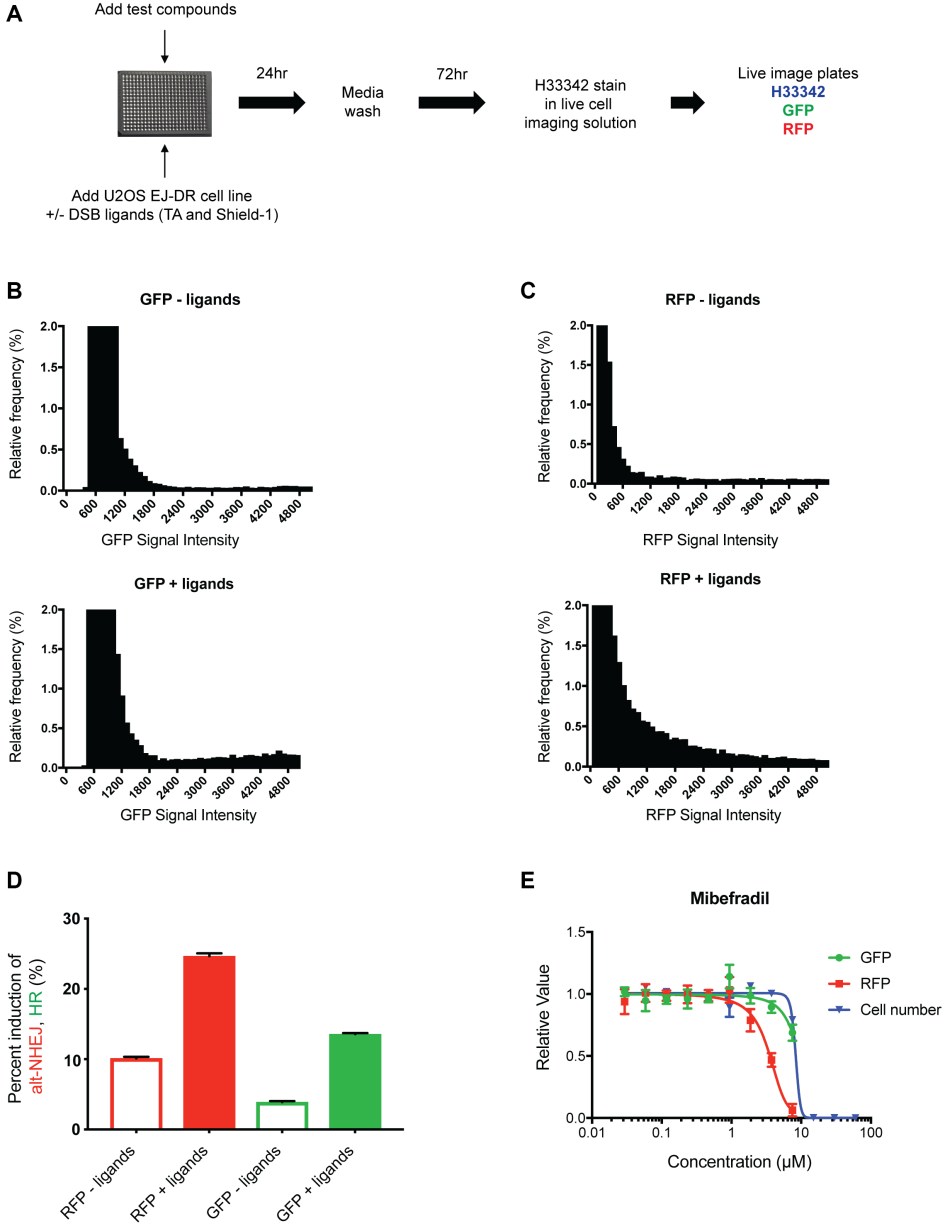
Development of the 384-well live imaging-based EJ-DR assay. (**A**) Schematic showing the workflow of the 384-well EJ-DR assay in the U2OS cell line. DSBs were induced using the ligands Triamcinolone and Shield-1 and a no ligand control plate was also included to measure baseline DNA repair. DSB induction was halted 24 hours after addition and 72 hours later the plates were imaged in the DAPI, GFP, RFP channel while cells were maintained in live imaging solution. (**B**, **C**) The nuclear GFP intensity (measure of HR) and RFP intensity (measure of alt-NHEJ) of each cell both with and without ligand addition was measured and plotted as a histogram. The no ligand control allowed for thresholding out baseline GFP and RFP activation. (**D**) The percent induction of GFP (~3.5-fold) and RFP (~3-fold) by DSB ligand addition. (**E**) Mibefradil shows selective inhibition of alt-NHEJ over HR without significant cellular cytotoxicity. Data are represented as mean ± SEM.

An overview of the workflow of this assay is presented in Figure 1A, which starts with the addition of the test compounds and DSB ligands, followed by a media wash 24 hours later to terminate DSB induction, and then live cell imaging 96 hours later. After live cell imaging, we quantify the individual intensities of GFP and RFP signal in cells using the IN Cell imager. We then use the intensity measurements of individual cells in each channel and plot these intensities as a histogram to threshold out baseline levels of GFP and RFP intensity in the absence of ligands (Figure 1B and 1C). After applying the threshold, the percent of GFP and RFP-positive cells is calculated as a readout of induction of HR and alt-NHEJ activity, respectively. As expected, we observe an increase in DNA damage and thus DNA repair upon DSB ligand addition, reflected as an increase in both GFP- and RFP-positive cells (Figure 1D).

Various doses of mibefradil were tested in this imaging-based EJ-DR assay to validate assay performance. Consistent with previous studies, mibefradil selectively inhibited alt-NHEJ with an IC50 of approximately 4 μM, confirming that the assay accurately detects and quantifies the effects of the drug. In addition to quantifying HR and alt-NHEJ activities, cytotoxicity in the absence of DSB ligands (CT) was also evaluated by quantifying the number of Hoeschst 33342-stained cells, allowing us to better understand the margin between on-target and presumable off-target, DNA repair-independent effects. IC50 values of HR inhibition, alt-NHEJ inhibition, and CT were used to calculate the “selectivity index” (SI) (defined as the ratio of CT IC50/alt-NHEJ IC50) and alt-NHEJ selectivity defined as HR IC50/alt-NHEJ IC50. We demonstrated that mibefradil showed expected patterns of selective alt-NHEJ inhibition over HR as previously described, i.e., alt-NHEJ IC50 ~ 4 μM, HR IC50 > 60 μM, SI = > 15, validating the robustness of this system to screen mibefradil analogues [10] (Figure 1E).

### Key structure-activity relationship findings of mibefradil

**Figure 2:**
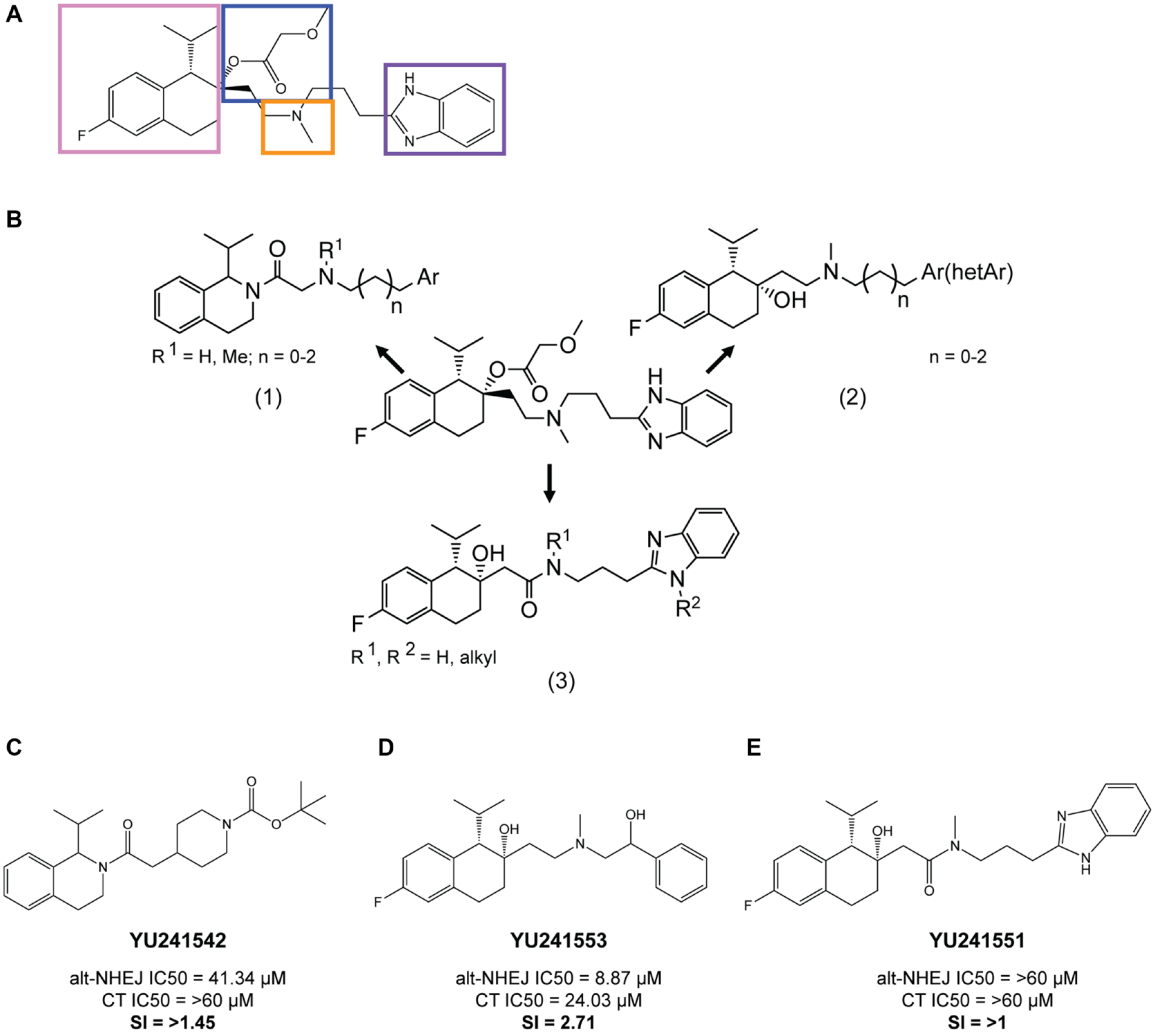
Key structure-activity relationship (SAR) findings of mibefradil. (**A**) Mibefradil pharmacophore showing the four main moieties, the substituted tetrahydronaphthalene core (pink), the tertiary amine linker (orange), benzimidazole ring (purple), and the methoxyacetate group (blue). (**B**) Overview of the three synthesis strategies (1) Replacement of the tetrahydronaphthalene group with a tetrahydroisoquinoline moiety, (2) Replacement of the benzimidazole moiety with other heterocyclic aromatic groups, (3) Replacement of the basic amine linkage with an amide linkage. (**C**) Analogue YU241542, a tetrahydroisoquinoline derivate, showed a dramatic reduction in alt-NHEJ inhibition IC50 to 41.34 μM and a selectivity index (SI) the ratio of cytotoxicity IC50 (CT IC50) to alt-NHEJ IC50 of 1.45. (**D**) Analogue YU241553, a derivative with a benzimidazole replacement, still demonstrated alt-NHEJ activity with an alt-NHEJ IC50 of 8.87 μM and SI of 2.71. (**E**) Analogue YU241551, an amide derivative, showed complete lack of alt-NHEJ activity with an alt-NHEJ IC50 > 60 μM and an SI of > 1.

Structure-activity relationship (SAR) studies were performed to identify structural elements of mibefradil essential for its alt-NHEJ inhibition and CCB function. The SAR studies focused on modifications to the four major moieties in mibefradil, the substituted tetrahydronaphthalene core (pink), the tertiary amine linker (orange), benzimidazole ring (purple), and the methoxyacetate group (blue) (Figure 2A). Mibefradil potently inhibits T-type calcium channels (Ca_v_3.1, Ca_v_3.2, Ca_v_3.3) but inhibits L-type calcium channels (Ca_v_1.2) with much lower potency [18]. The methoxyacetate group has previously been shown to be responsible for the calcium channel blocking ability of mibefradil, with deacetylated analogs showing greatly reduced calcium channel inhibition [19, 20]. We hypothesized that if the methoxyacetate group was critical for alt-NHEJ inhibition, its hydrolysis would also reduce the alt-NHEJ activity, and synthesized several deacylated analogues of mibefradil to test this hypothesis. Table 1 shows three representative analogues and the fold change of their T-type and L-type calcium channel IC50 value with respect to mibefradil. As expected, the deacylated analogues showed decreased potency of T-type and L-type calcium channel inhibition ranging between a 3 to 17-fold decrease. However, the deacylated analogues of mibefradil showed either equipotent or more potent inhibition of alt-NHEJ activity, suggesting that DNA repair activity of mibefradil is uncoupled from its T-type and L-type calcium channel blocking activities. This prompted us to explore further modifications to deacylated mibefradil.

**Table 1:** The DNA repair activity of mibefradil is uncoupled from its calcium channel blocking activity

IC50, μM	Mibefradil	YU252386	YU252377	YU252222
nc-EJ	3.8	1.1	1.5	5.8
HR	> 60	> 60	> 60	> 60
Cytotoxicity	10.5	17.9	14.8	> 60
L type Cav 1.2	6.6	5.6	6.3	> 30
T type Cav 3.1	1.1	0.9	5.3	11.1
T type Cav 3.2	1.1	0.9	5.6	11.8
T type Cav 3.3	1.3	1.1	8.2	12.9

Table 1 shows three representative deacetylated mibefradil analogues and their corresponding IC50 values for nc-EJ, HR inhibition, cytotoxicity, and their calcium channel blocking ability compared to mibefradil.

To determine whether the other three moieties were responsible for alt-NHEJ inhibition, we synthesized three broad categories of analogues: (1) Replacement of the tetrahydronaphthalene group with a tetrahydroisoquinoline moiety, (2) Replacement of the benzimidazole moiety with other heterocyclic aromatic groups, (3) Replacement of the basic amine linkage with an amide linkage (Figure 2B). Representative analogues from each synthesis strategy are shown in Figure 2C, 2D and 2E along with their corresponding alt-NHEJ inhibition IC50, cytotoxicity IC50, and selectivity index (SI) calculated as the ratio of cytotoxicity IC50 and alt-NHEJ inhibition IC50. Tetrahydroisoquinoline derivatives showed a dramatic reduction in alt-NHEJ inhibition in comparison with mibefradil, while replacing the benzimidazole ring did not greatly affect alt-NHEJ inhibition (alt-NHEJ IC50 = 8.87 μM). However, analogues with an amide linkage were entirely inactive in inhibiting alt-NHEJ (alt-NHEJ IC50 > 60 μM). The key SAR findings include (1) the tetrahydronaphthalene core and tertiary hydroxyl group is essential for alt-NHEJ inhibition as all five synthesized tetrahydroisoquinoline derivatives (data not shown) showed lack of alt-NHEJ activity, (2) The benzimidazole moiety is not essential for alt-NHEJ inhibition as all three synthesized aromatic benzimidazole replacements (data not shown) still retained alt-NHEJ activity, (3) The basic tertiary amine is important for maintaining activity as all four amide linked synthesized analogues (data not shown) were inactive. Given these key SAR findings, we then moved to reducing hERG inhibition while keeping the tetrahydronaphthalene and basic linker amine core intact.

### Synthesis of mibefradil analogues with reduced hERG activity

hERG is a potassium ion channel responsible for repolarization of the cardiac action potential. Inhibition of hERG blocks the repolarization of cardiac cells, which leads to QT prolongation and can predispose individuals to arrhythmias [21]. In our mibefradil phase I trial, five out of 19 patients showed QT prolongation (manuscript under preparation, [12]). We first evaluated the hERG inhibition by mibefradil using a patch clamp hERG assay and determined its IC50 to be 1.5 μM, which overlaps with its alt-NHEJ activity. Our overall goal was to reduce the hERG activity by 5 to 10-fold while maintaining alt-NHEJ activity. To modulate the hERG inhibition associated with mibefradil, we developed a rational drug design approach based on previous studies of classical hERG inhibitors [22, 23]. These reports demonstrated that most hERG inhibitors shared the following structural similarity, a basic nitrogen center linked through flexible linkers to two aromatic groups. However, we had previously determined that the tertiary amine in mibefradil was critical for its alt-NHEJ function.

**Figure 3:**
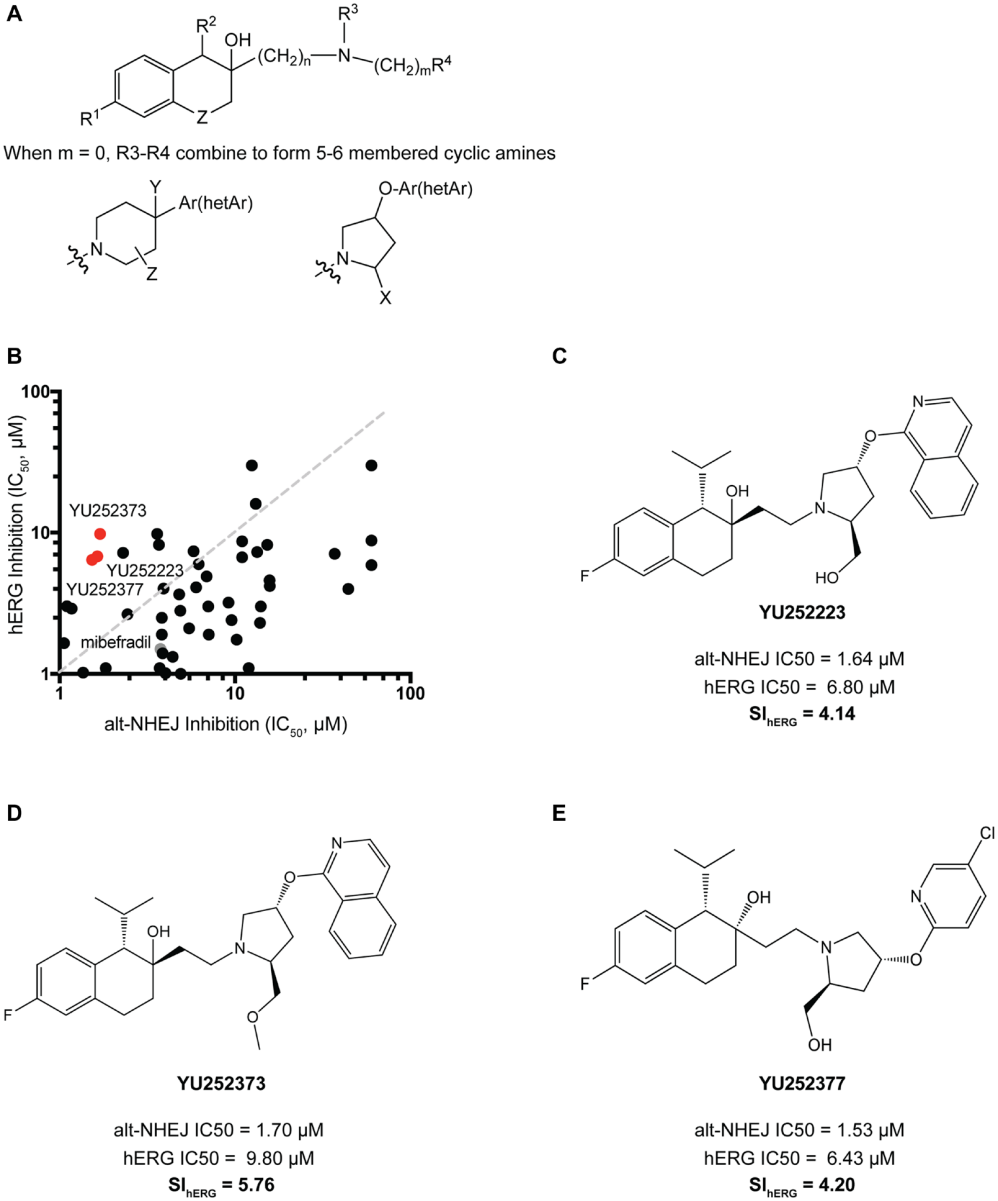
Synthesis and validation of mibefradil analogues with reduced hERG activity. (**A**) Mibefradil derivative showing the overall strategy to reduced hERG inhibition where R1 = H, halogen, alkoxy, CF3, OCF3, OCH2CF3; R2 = H, alkyl, cycloalkyl; R3 = OH, OC(O)R, OCO2R, OC(O)CH2OR, OC(O)NR2 where R = alkyl or cycloalkyl; R4 = alkyl or cycloalkyl, CH2CF3, aryl, benzyl, alkoxy; *n* = 1–3 and m = 0–3; R5 = Phenyl, phenyl substituted with halogen, alkoxy, CF3, OCF3, OCH2CF3 or R5 = pyridine, isoquinoline, pyrazine, pyridazine, indazole also substituted with halogen, alkoxy, CF3; Z = CH2, CF2, O, S; when m = 0, R4 and R5 combined to represent cyclic amines where Ar = Phenyl or Phenyl group substituted with halogen, alkoxy, CF3, OCF3, OCH2CF3; hetAr = pyridine, isoquinoline, pyrazine, pyridazine group substituted with halogen, alkoxy, CF3; X = CH2OR, CO2R where R = H, lower alkyl, cycloalkyl; Y = H, OH, alkoxy, CO2R where R = H, lower alkyl, benzyl; Z = H, OH, halogen. (**B**) 48 analogues with a reduced basicity of the tertiary amine and increased lipophilicity of their side groups were screened for their alt-NHEJ IC50 vs their hERG IC50. The top hits YU252373, YU252223, and YU252377 are indicated in red and mibefradil in gray. (**C**) Analogue YU252223 showed a 4.14-fold reduction in hERG potency calculated as the selectivity index (SI_hERG_) of hERG IC50/alt-NHEJ IC50. (**D**) Analogue YU252373 showed a 5.76-fold reduction in hERG potency. (**E**) Analogue YU252377 showed a 4.20-fold reduction in hERG potency.

As such, we aimed to reduce the basicity of the tertiary amine and increase lipophilicity of the side groups as summarized in Figure 3A. We synthesized 48 analogues with modifications to the aromatic side rings and the tertiary amine. To increase the lipophilicity of the aromatic side groups, bulky alkyl and aryl substituents were added in addition to fluoro-substituted alkyl groups. To reduce the basicity of the tertiary amine, electron-withdrawing substituents were added and five or six-membered heterocyclic rings were also explored. All 48 analogues were profiled for their hERG activity using a patch clamp hERG assay and alt-NHEJ inhibition (Figure 3B). Figure 3C, 3D and 3E shows the top three analogues which demonstrated markedly reduced hERG inhibition (~5 fold), while retaining potent alt-NHEJ activity, represented as selectivity index for hERG (SI_hERG_), or the ratio of hERG IC50 to the alt-NHEJ IC50. Notably, all three analogues contained a five-membered heterocyclic pyrrolidine, which suggests that reducing the basicity of the tertiary amine is a viable approach to reducing hERG inhibition. Following the synthesis of mibefradil analogues with reduced hERG activity, we undertook a similar rational drug design approach to synthesizing mibefradil analogues with reduced CYP inhibition.

### Synthesis of mibefradil analogues with reduced CYP3A4 and CYP2D6 activity

Mibefradil was previously FDA approved for hypertension and then withdrawn owing to potent drug-drug interactions with a wide range of drugs including β-blockers, simvastatin, digoxin, methylprednisolone, diltiazem, and cyclosporin leading to severe toxicities including cardiogenic shock, myopathy, renal failure, and death [13, 14, 24–26]. These interactions were largely due to the potent inhibition of the cytochrome P450 enzymes (CYPs) by mibefradil. CYPs are oxidative enzymes primarily located in liver cells and responsible for the clearance of the majority of drugs. Specifically, mibefradil has been shown to inhibit CYP3A4 and CYP2D6, responsible for the metabolism of roughly 60% and 25% of drugs [27, 28]. This inhibition is largely through mechanism-based inhibition, where the metabolite of mibefradil irreversibly binds to CYP3A4 [29, 30]. Using human liver microsomes, we measured the *in vitro* inhibition of both CYP enzymes by mibefradil. Our data confirmed that mibefradil is a potent inhibitor of both CYP3A4 (IC50 = 0.8 μM) and CYP2D6 (IC50 = 0.6 μM). Our aim was to reduce activity against CYPs using rational drug design and previous SAR information based on common CYP inhibitors, while retaining the alt-NHEJ activity of the analogues [31, 32]. Previous SAR studies had described potential interacting sites of mibefradil with CYP3A4 and CYP2D6. These include polar interactions through the central amine, and hydrophobic interactions through the benzimidazole ring and tetrahydronaphthalene core with aromatic groups close to the active site of the enzyme [33, 34].

**Figure 4:**
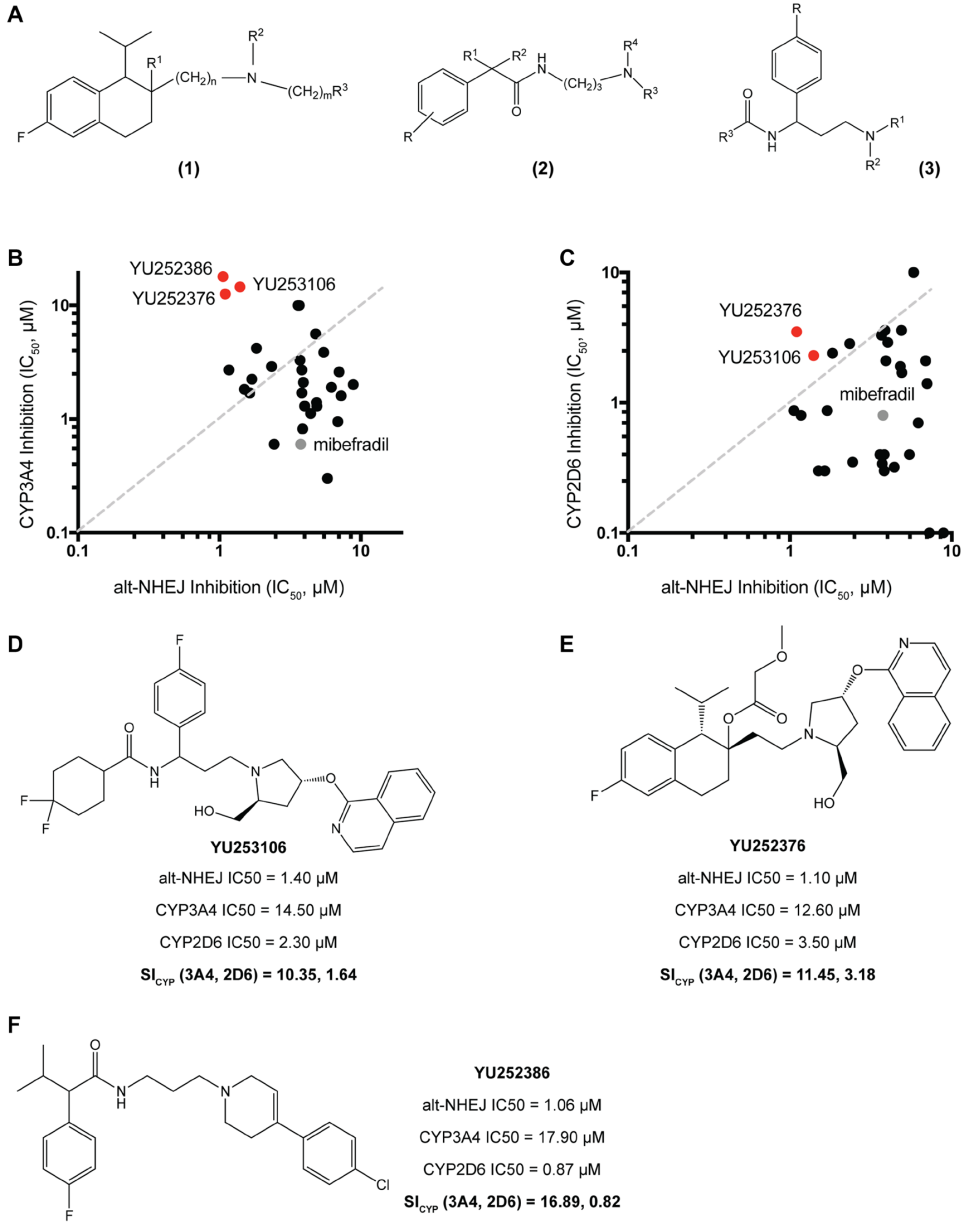
Synthesis and validation of mibefradil analogues with reduced CYP3A4 and CYP2D6 activity. (**A**) Three main synthesis strategies were used to reduce CYP inhibition (1) Retaining the tetrahydronaphthalene core while modifying the basicity of the central amine or reducing the lipophilicity of aromatic side groups: R1 = OH, OC(O)R, OCO2R, OC(O)CH2OR, OC(O)NR2; R2 = alkyl or cycloalkyl, CH2CF3, aryl, benzyl, alkoxy; *n* = 1–3 and m = 0–3; R3 = Phenyl, phenyl substituted with halogen, alkoxy, CF3, OCF3, OCH2CF3 or R3 = pyridine, isoquinoline, pyrazine, pyridazine, indazole also substituted with halogen, alkoxy, CF3; when m = 0, R4 and R5 combined to represent cyclic amines. (2), (3) Modifying the tetrahydronaphthalene core to explore alternate scaffolds: R = H, alkyl, OH; R1 = alkyl, acyl, SO_2_Me, heteroaryl; R2 = H, alkyl. (**B**) 29 analogues were screened for their alt-NHEJ IC50 vs their CYP3A4 IC50 with the top hits YU252376, YU252386, and YU253106 indicated in red and mibefradil in gray. (**C**) 29 analogues were screened for their alt-NHEJ IC50 vs their CY2D6 IC50 with the top hits YU252376, and YU253106 indicated in red and mibefradil in gray. (**D**) Analogue YU253106 showed a decrease in CYP3A4 and CYP2D6 IC50 by 10.35 and 1.64-fold respectively represented as the selectivity index of CYP (SI_CYP_). (**E**) Analogue YU252376 showed a decrease in CYP3A4 and CYP2D6 IC50 by 11.45 and 3.18-fold respectively represented as the selectivity index of CYP (SI_CYP_). (**F**) Analogue YU252386 showed a decrease in CYP3A4 and CYP2D6 IC50 by 16.89 and 0.82-fold respectively.

To modulate the CYP3A4 and CYP2D6 activity, we synthesized 29 analogues using the following two strategies: (1) Retaining the tetrahydronaphthalene core while modifying the basicity of the central amine or reducing the lipophilicity of aromatic side groups, and (2) Modifying the tetrahydronaphthalene core to explore alternate scaffolds. For the first approach, we synthesized analogues using a similar scaffold as that in Figure 3A, which followed a similar rationale (Figure 4A (1)). For the second approach, we took advantage of our previously published high-throughput screen of FDA approved drugs, which lead to the identification of mibefradil and other notable hits such as loperamide, an anti-diarrheal, and proadifen, a non-selective CYP inhibitor [10]. These drugs showed a similar phenotype and structure to mibefradil, i.e., selective inhibition of alt-NHEJ, but different pharmacology. To explore modifications to the tetrahydronaphthalene core, we synthesized loperamide and proadifen analogues (Figure 4A (2), (3)). All 29 analogues were profiled for their CYP3A4 and CYP2D6 activity *in vitro* using human liver microsomes along with their alt-NHEJ inhibition (Figure 4B, 4C). Figure 4D, 4E and 4F show the top three analogues which demonstrated the greatest reduction in activity against CYP3A4 and CYP2D6 while retaining potent alt-NHEJ activity, represented as their selectivity index for CYP (SI_CYP_). All three analogues showed a >10-fold decrease in CYP3A4 inhibition while YU252376 showed a ~3-fold decrease in CYP2D6 inhibition. Our data demonstrates that the methoxyacetate group may not be as critical to CYP inhibition as previously thought, compared to the benzimidazole ring, as YU252376 showed meaningful reduction in CYP inhibition with modifications to only the amine group and aromatic side chains [27]. After successfully reducing the CYP activity of mibefradil analogues, we went on to studying the *in vitro* and *in vivo* pharmacokinetics of the optimized compounds.

### Evaluating pharmacokinetic parameters for the most promising mibefradil analogues

**Figure 5:**
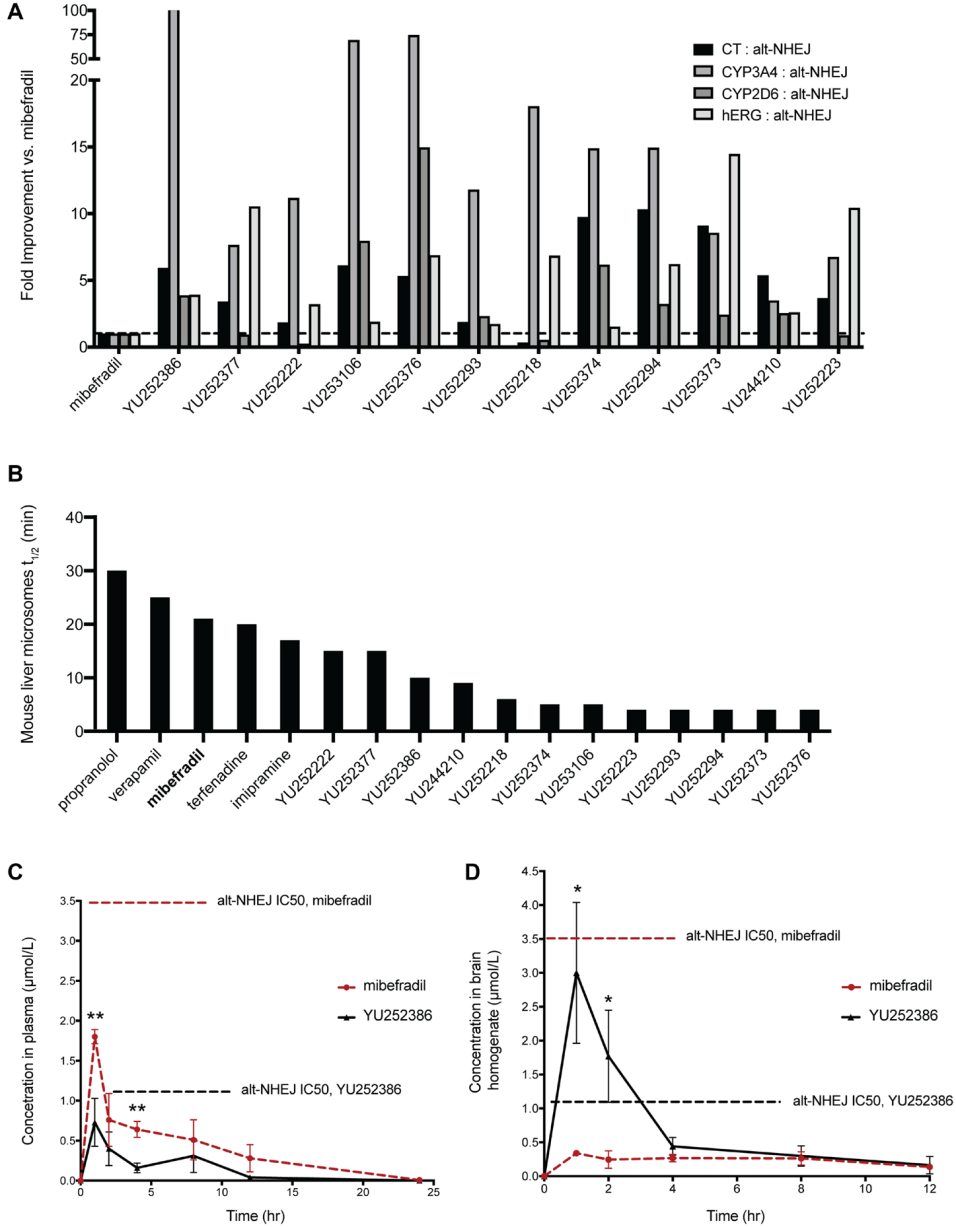
Evaluating the pharmacokinetic profile of the top 12 analogues of mibefradil. (**A**) Fold improvement of the top 12 mibefradil analogues over mibefradil in terms of their cytotoxicity IC50, CYP3A4 IC50, CYP2D6 IC50, and hERG IC50 compared to their alt-NHEJ IC50. (**B**) The mouse liver microsomal half-life (t_1/2_) of the 12 mibefradil analogues compared to mibefradil and four FDA-approved drugs known to be metabolized in liver microsomes. (**C**) Plasma concentration of mibefradil and YU252386 dosed orally at 30 mg/kg in female athymic nude mice over 24 hours (*n* = 3). The *in vitro* alt-NHEJ IC50 of both mibefradil and YU252386 is indicated on the graph. (**D**) Brain homogenate concentration of mibefradil and YU252386 dosed orally at 30 mg/kg in female athymic nude mice over 24 hours (*n* = 3). Data are represented as mean ± SEM.

Following studies to reduce the off-target activities of mibefradil analogues, we selected 12 analogues for further evaluation. These analogues showed improved potency, reduced cytotoxicity, and reduced off-target effects compared to mibefradil (Figure 5A). The overall goal was now to identify lead compounds for future *in vivo* studies. We first tested the metabolic stability of the selected analogues in mouse liver microsomes. Phase I metabolic enzymes in liver microsomes account for ~80% of total drug metabolism. Testing the half-lives of the analogues incubated with liver microsomes allows for the estimation of the anticipated plasma exposure of the drug and its intrinsic clearance. Those with a lower half-life would be expected to attain a lower steady-state plasma concentration compared with an equipotent drug with a higher half-life. We profiled the mouse liver microsome half-life of mibefradil, twelve analogues, and previously FDA-approved drugs which are known to be metabolized in these microsomes i.e., Propranolol, Verapamil, Terfenadine, and Imipramine (Figure 5B). Mibefradil has a half-life of 15 minutes, and multiple analogues showed a similar or slightly lower half-life. All 12 analogues were ranked according to the ratio of their cytotoxicity, hERG, CYP3A4, and CYP2D6 IC50 to their alt-NHEJ IC50 and their estimated microsomal half-life. Following this ranking, the top three hits, namely YU252222, YU252377, and YU252386, were selected for an *in vivo* pharmacokinetic study.

Female athymic nude mice were orally dosed with 30 mg/kg of YU252222 (a methyl ester prodrug was used), YU252377 and YU252386, and plasma and brain concentration were recorded at 1, 2, 4, 8, 12, 24 hours post dosing. These measurements were used to calculate various pharmacokinetic parameters critical to bioavailability including time to reach maximum concentration (t_max_), maximum concentration achieved (C_max_), and half-life (t_1/2_) (Figure 5C, 5D, Supplementary Table 1). YU252222 and YU252377 had very limited plasma exposure (C_max_ << 100 ng/mL) and did not achieve detectable brain exposure. Notably, YU252386 showed improved brain exposure compared to mibefradil (~8-fold) and this concentration was greater than the alt-NHEJ IC50 (~3-fold), suggesting that this dose could inhibit alt-NHEJ in the brain. Correspondingly, YU252386 showed a markedly reduced concentration in the plasma compared with mibefradil (~0.3-fold), which could imply specificity to brain tissue. YU252386 showed a half-life of ~2.5 hours while mibefradil showed a half-life of ~8 hours in the brain tissue. This suggests that YU252386 could require more frequent dosing to achieve a meaningful and sustained inhibition of alt-NHEJ. However, given that the percent coverage of the *in vitro* alt-NHEJ IC50 of YU252398 at the C_max_ in the brain is 300%, and this analogue is highly brain penetrant, this may not prove to be a shortcoming for future *in vivo* studies. The results so far demonstrate the successful identification of a novel analogue of mibefradil, YU252386, which shows improved potency, reduced off-target effects, and increased specificity towards the brain *in vivo*.

### Identification of potential regulators of the selective alt-NHEJ inhibition of mibefradil

**Figure 6:**
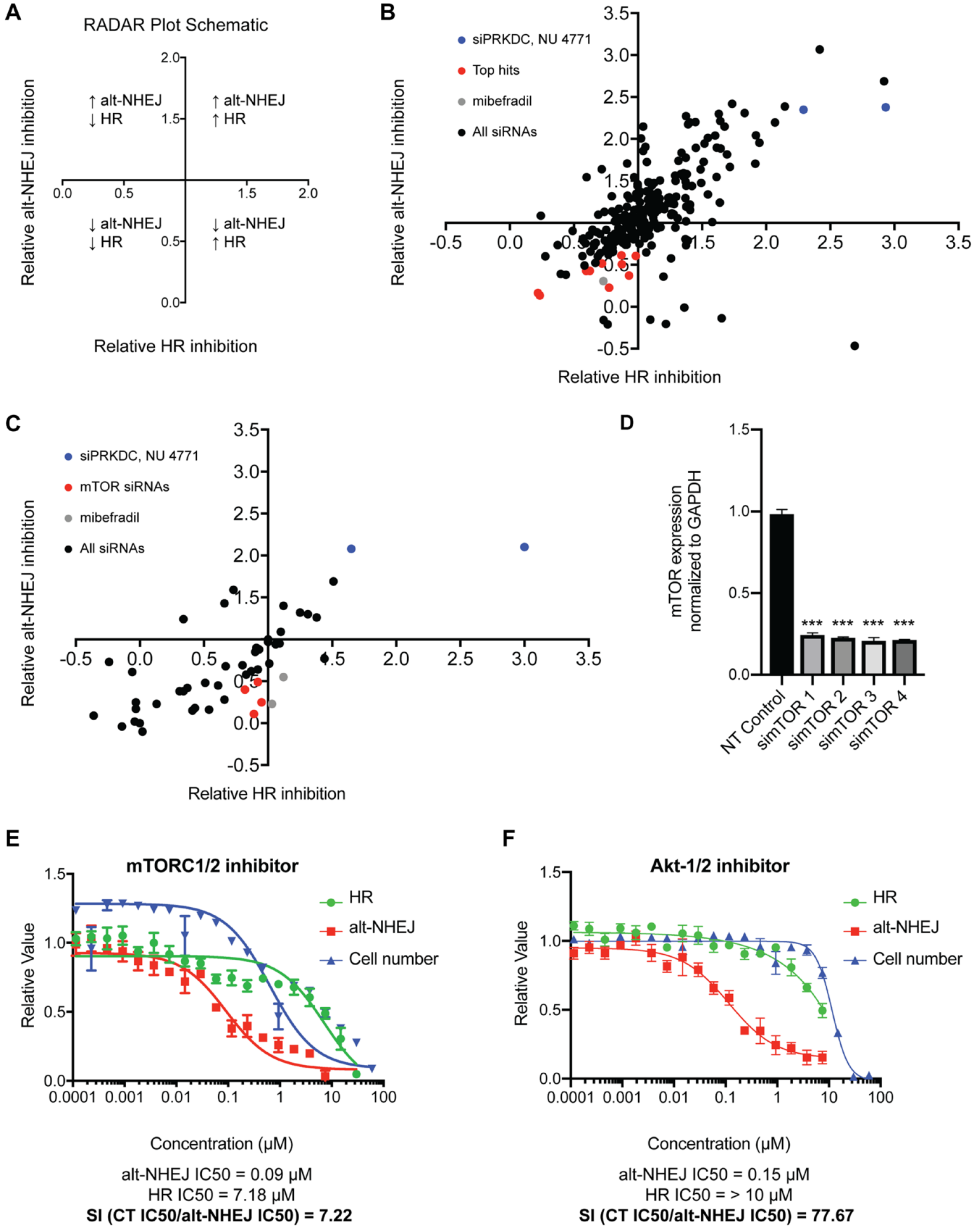
Identification of potential regulators of the selective alt-NHEJ inhibition of mibefradil. (**A**) A schematic representation of the Relative Assessment of DNA Repair (RADAR) plot showing the significance of the different quadrants and their correlation with DNA repair activity. (**B**) An siRNA screen of 240 siRNAs against common DDR proteins in the EJ-DR assay showing the top hits phenocopying mibefradil in red, the controls siPRKDC (DNA-dependent protein kinase catalytic subunit) and 2 μM NU 4771 (DNA-PK inhibitor) in blue, and 3 μM and 6 μM mibefradil in gray. (**C**) A focused screen of the deconvoluted siRNAs for the top eleven hits identified in the previous screen in the EJ-DR assay. The top hit simTOR is indicated in red, the controls siPRKDC (DNA-dependent protein kinase catalytic subunit) and 2 μM NU 4771 (DNA-PK inhibitor) in blue, and 3 μM and 6 μM mibefradil in gray. (**D**) Deconvoluted siRNAs to mTOR reduced mRNA expression of mTOR to ~20% of the non-targeting (NT) control. Gene expression is normalized to a GAPDH control. (*n* = 3, ^***^ *p* < 0.001 by Student’s *T* test) (**E**) Dose-response curve of mTORC1/2 inhibitor (mTORC1/2i) in the EJ-DR assay showing alt-NHEJ activity in red, HR activity in green, and cytotoxicity (CT) in blue. mTORC1/2i showed a selectivity index (SI), calculated as CT IC50/alt-NHEJ IC50, of 7.22. (**F**) Dose-response curve of Akt1/2-inhibitor (Akt-1/2i) in the EJ-DR assay. Akt-1/2i showed an SI of 77.67. Data are represented as mean ± SEM.

To try to understand the potential target and the mechanism of selective alt-NHEJ inhibition by mibefradil and its analogues, we adapted the imaging-based high-throughput EJ-DR assay to screen a custom siRNA library targeting 240 different DNA repair proteins. The ON-Target SMARTpool siRNA DNA repair library was used to knockdown 240 individual DNA repair proteins to assess which proteins phenocopied the selective inhibition shown by mibefradil. Cells were transfected with siRNAs from the DDR library and 72 hours after transfection, DSB ligands and control compounds were added. 96 hours after DSB induction, cells were imaged and relative GFP/RFP intensity and cell viability were determined. The change in GFP/RFP intensity was used to calculate the relative inhibition of alt-NHEJ and HR for each siRNA compared to the RISC-free control. To visualize the results of this screen, we utilized a Relative Assessment of DNA Repair (RADAR) plot, the schematic for which is shown in Figure 6A, to group siRNAs into different quadrants based on their modulation of either alt-NHEJ or HR (Figure 6B). siPRKDC (DNA-dependent protein kinase catalytic subunit) and NU 4771 (DNA-PK inhibitor) were used as controls (in blue; Figure 6B). Knockdown and inhibition of this key DDR protein caused an increase in both alt-NHEJ and HR as previously reported [9]. Mibefradil (in gray) was also tested, which caused the selective inhibition of alt-NHEJ over HR as expected. This screen revealed a number of previously known and unknown hits involved in the selective regulation of HR and alt-NHEJ. Critical HR genes including BRCA1, BRCA2, RAD51, and PALB2 showed the greatest HR/alt-NHEJ specificity, as expected (data not shown). Additionally, POLQ, a critical regulator of alt-NHEJ, also showed a 1.17-fold selectivity (defined as ratio of relative HR inhibition to relative alt-NHEJ inhibition) for alt-NHEJ over HR (Supplementary Figure 1).

The criteria for choosing hits from the DNA repair screen included: (1) Inhibition of either alt-NHEJ and HR with a relative value of alt-NHEJ and HR inhibition < 1, (2) Greater inhibition of alt-NHEJ compared to HR, i.e., a ratio of inhibition of alt-NHEJ to HR inhibition < 0.8, (3) Cell viability greater than 80%, i.e., cytotoxicity > 0.8. Top hits included RPA2, POLA, DDB1, TYMS, CHEK1, mTOR, TTRAP, FEN1, UBE2A, PARP3, and ATRX.

We followed up the DDR screen with a focused deconvolution screen where four siRNAs comprising SMARTpool in the primary screen were tested individually for the top eleven hits (Figure 6C). mTOR (in red) was the only hit that showed consistent selective alt-NHEJ inhibition over HR across all four deconvoluted siRNAs. Additionally, we also validated that the four individual mTOR siRNAs caused mTOR knockdown using RT-qPCR (Figure 6D). Each of the individual mTOR siRNAs caused an ~80% decrease in mTOR gene expression. We also tested three potent mTOR inhibitors in the EJ-DR assay, which also phenocopied mibefradil and showed selective inhibition of alt-NHEJ over HR (Figure 6E, Supplementary Table 2). We then interrogated downstream kinases of mTOR including S6K, CDK4/6, and CHK1/2. Neither S6K nor CHK1/2 inhibitors showed an activity in the EJ-DR assay. The CDK4/6 inhibitor showed selective inhibition of HR over alt-NHEJ, which suggests that there is no involvement of these kinases in the selective regulation of alt-NHEJ by mTOR (Supplementary Table 2). To further understand which DDR proteins could potentially be involved in this alt-NHEJ regulation, we tested inhibitors of other upstream regulators of the DDR including ATM, ATR, PI3K/Akt, PTEN. Akt inhibitors showed an exquisite sensitivity (~80-fold) towards alt-NHEJ over HR. We tested five Akt inhibitors and all five phenocopied mibefradil (Figure 6F, Supplementary Table 2). Taken together, we determined that both mTOR and Akt are involved in the regulation of alt-NHEJ and may be involved in the selective regulation of alt-NHEJ by mibefradil.

## DISCUSSION

We report the synthesis and validation of novel analogues of the potent T-type and L-type CCB, mibefradil, for potential use as radiosensitizers in GBM. Radiation induces both single-strand breaks (SSBs), double-strand breaks (DSBs), as well as a number of complex lesions which require HR for their repair. Alt-NHEJ functions as a back-up pathway for HR for the repair of these complex lesions and DSBs, which, if unrepaired, can be lethal to the cell. We previously reported that mibefradil showed potent and selective inhibition of alt-NHEJ over HR. However, while mibefradil was previously FDA approved for use in hypertension, it was subsequently withdrawn from the market owing to multiple off-target effects including hERG channel inhibition leading to QT prolongation and arrhythmias as well as inhibition of CYP enzymes leading to severe drug-drug interactions. To minimize these off-target effects, while retaining DNA repair inhibition activity, we developed novel SAR approaches and reported the synthesis of over 140 mibefradil analogues. We developed a 384-well imaging-based EJ-DR assay to rapidly screen the DNA repair activity of the synthesized analogues, and validated the results of this assay using mibefradil and NU 7441 as controls. This assay also closely recapitulated the results of the original flow cytometry-based EJ-DR assay and as such, represents an improvement on the previous assay to screen a larger library of compounds more rapidly.

Following the development of this assay, we performed preliminary SAR studies to determine which moieties were critical for the alt-NHEJ inhibition by mibefradil. We determined that the tetrahydronaphthalene core and tertiary amine in mibefradil contributed to its alt-NHEJ function, while the benzimidazole ring and the methoxyacetate group (responsible for the CCB ability), were non-essential. This was an important finding, which could also aid the development of more selective CCB analogues. Mibefradil has both T-type and L-type CCB activity, however it is still unclear which calcium channel is crucial for its unique cardiovascular effects. The analogue YU252377 showed only a slight reduction in its L-type blocking over mibefradil while a > 10-fold decrease in T-type channel activity (Figure 2B). Testing such L-type specific analogues in cardiomyocytes and performing additional SAR studies, could further our understanding of the anti-hypertensive effects of mibefradil.

After determining preliminary SAR, we began to further understand the structure-liability relationship (SLR) with the mibefradil pharmacophore. Using the structures of common hERG inhibitors and other previously established SAR approaches, we synthesized 48 analogues with increased lipophilicity of the aromatic side chains and reduced basicity of the central amine. We were able to reduce hERG inhibition by ~5-fold, while retaining alt-NHEJ activity. While this was a significant improvement over the highly potent hERG inhibition by mibefradil, a report in 2003 suggested that a 30-fold margin between the C_max_ and hERG inhibition IC50 of compounds in pre-clinical development would allow for a reduction in the hERG-associated cardiotoxicity [35]. Prior to xenograft studies using the mibefradil analogues, *in vivo* hERG inhibition studies may be required to validate the reduction in this off-target effect. Further modifications to the aromatic side chains and the central amine of the synthesized analogues may be required to widen the therapeutic margin. We undertook a similar approach to minimize the CYP3A4 and CYP2D6 inhibition of mibefradil and synthesized 29 analogues with >10-fold reduction in CYP3A4 activity and ~3-fold reduction in CYP2D6 activity. The syntheses strategies we employed for reducing CYP inhibition included modifications to the tetrahydronaphthalene core, which have been previously unexplored in SAR studies with mibefradil. We utilized alternate scaffolds adapted from the structures of loperamide and proadifen, which we had previously identified as selective alt-NHEJ inhibitors [9]. This approach proved largely successful as both YU252386 was synthesized using this alternative template and showed dramatic reductions in CYP3A4 inhibition (~17-fold).

After successfully reducing the known off-target liabilities of mibefradil, we selected the top 12 analogues for further *in vitro* and *in vivo* pharmacokinetic studies. YU252386 showed the greatest improvement in pharmacokinetic profile over mibefradil including an ~8-fold increase in brain exposure and a ~0.3-fold decrease in plasma exposure. These pharmacokinetic attributes make it the most suitable for future *in vivo* radiosensitization studies. Prior to performing such studies, it will be critical to determine optimal timing and dosing regimens to achieve sufficient brain tissue penetration of the compound before administering radiotherapy.

To determine the critical proteins involved in this unique regulation of alt-NHEJ, we performed a high-throughput siRNA screen to determine which DDR proteins phenocopied the DNA repair activity of mibefradil. Interestingly, siRNAs targeting POLQ, the critical alt-NHEJ gene, showed a specificity for alt-NHEJ over HR. All four siRNAs targeting mTOR also showed a selective inhibition of alt-NHEJ over HR. After validating the knockdown of mTOR, we also tested three mTOR inhibitors, AZD 3147 (mTORC1/2i), AZD 8055 (mTORi), and Everolimus (mTORC1i), in the EJ-DR assay, which showed similar results. We then interrogated kinases downstream of mibefradil including S6K, CDK4/6, and CHK1, however, knockdown or small molecule inhibition of these targets did not explain the alt-NHEJ activity of mTOR. After testing the knockdown of a number of proteins in the DDR pathway, we discovered that knockdown and inhibition of Akt closely phenocopied mibefradil and mTOR. Previous reports have presented a contradictory view on the regulation of the Akt pathway by mibefradil with one report describing the activation of PI3K/Akt/mTOR by mibefradil while another report demonstrated mibefradil reduced the phosphorylation of Akt [36, 37]. This suggests that there may be context-specific roles of Akt regulation by mibefradil, which warrants further study. Additionally, there have been multiple reports about the role for the three isoforms of Akt in the positive and negative regulation of both HR and NHEJ in response to chemotherapy and other DNA damaging agents [38–41]. However, a role for Akt in alt-NHEJ has not been previously published. Similarly, the role of mTOR in either HR or NHEJ pathway has been previously documented, owing to the close overlap between proteins in the mTOR pathway and the DDR pathways [42, 43]. However, limited reports exist about the link between mTOR and alt-NHEJ. It is also possible that the lack of an appropriate assay to account for the alt-NHEJ activity of mTOR/Akt confounded these results. The results reported above prompt the further investigation of the role of mTOR/Akt in the activation of alt-NHEJ.

The use of DNA repair inhibitors as radiosensitizers in GBM could represent a viable approach to achieve better response owing to the range of DDR pathways activated in response to radiation-induced DNA damage. Additionally, the identification of selective inhibitors of alt-NHEJ could also be tested in other settings where alt-NHEJ activity is critical, such as in HR-deficient tumors [44, 45]. The synthesis and validation of the mibefradil analogue, YU252386, shows great promise towards the development of a potent and selective radiosensitizer for GBMs and beyond, and warrants further *in vivo* study in clinically relevant GBM models.

## MATERIALS AND METHODS

### Cell lines and culture conditions

The EJ-DR cell line was created in the U2OS cells (a human osteosarcoma cell line), and contained a stably integrated EJ-RFP plasmid to measure alt-NHEJ repair and a DR-GFP plasmid to measure HR repair, and has been previously described [9]. The EJ-DR cell line was cultured in high glucose Dulbecco’s modified Eagle’s medium (DMEM; Thermo Fisher Scientific) with L-glutamine containing 10% tetracycline-free (tet-free) fetal bovine serum (FBS; Takara Bio Inc. and Atlanta Biologics) and 1% Penicillin Streptomycin (Thermo Fisher Scientific). Tet-free FBS was used to prevent any expression of the tetracycline-inducible EJ-RFP system from residual tetracycline found in most commercially available FBS. All cells were maintained at 37°C with 5% CO_2_.

### Calcium channel activity studies

Calcium channel blocking studies were performed by Charles River Laboratories in Chinese Hamster Ovary (CHO) and HEK293 cell lines stably expressing L-Type and T-type calcium channels, respectively, under tetracycline induction in accordance with a previously published study [46]. Data acquisition and analysis was performed using the IonWorks Quattro™ or Barracuda system software (Molecular Devices Corporation).

### Development of the EJ-DR 384-well plate assay

The EJ-DR cells were plated in 384-well imaging plates at 2500 cells/well. 24 hours later, the DSB ligands, Triamcinolone Acetonide (TA; Sigma-Aldrich Co., LLC) and Shield-1 (Takara Bio Inc.), responsible for inducing the DSBs, were added for a final concentration of 100 nM TA and 1 μM Shield-1. Test compounds were also added at the same time using Echo 550 Acoustic Dispenser (Labcyte). A separate plate was seeded to test for cytotoxicity of the test compounds in the absence of the DSB ligands. All EJ-DR experiments included the internal controls mibefradil (Tocris Bioscience) and DNA protein kinase catalytic subunit inhibitor (NU-7441, Cayman Chemical Company). The DSB ligands were washed out 24 hours after addition. 96 hours after addition of the ligands and test compounds, the media was washed off and cells were incubated with Hoechst nuclear stain in Live Cell Imaging Solution (Thermo Fisher Scientific). Plates were rinsed and prepared for immediate live cell imaging using GE IN Cell 2200 (Cytiva Life Sciences). Images were analyzed using InCell Analyzer software (Cytiva Life Sciences). Nuclei were segmented based on the Hoechst staining, and the intensity of the GFP and RFP signals in control populations were measured and binned into histograms to determine signal intensity threshold. The GFP and RFP intensity of all wells was then analyzed, and the percent of positive cells with GFP and RFP intensity above the threshold was calculated to determine the induction of signal. The DAPI cell counts were used to determine the relative cytotoxicity of test compounds in the absence of induced DNA damage.

### Synthesis of mibefradil analogues

Mibefradil analogues were generated as described in Supplementary Materials and Methods.

### Automated hERG inhibition patch clamp assay

Automated whole cell voltage clamp hERG inhibition assays were performed in Human hERG (Kv11.1) expressed in CHO-K1 cells optimized for automated patch clamp studies by Sophion Bioscience A/S [47].

### Cytochrome P450 inhibition assay

CYP inhibition assays were performed by Eurofins Scientific. Test compounds were incubated with human liver microsomes, followed by compound detection using high performance liquid chromatography with tandem mass spectrometry (HPLC-MS/MS) [48].

### *In vitro* and *in vivo* pharmacokinetic studies

Microsomal half-life studies were performed at Eurofins Scientific using mouse liver microsomes and test compounds were incubated for 0, 15, 30, 45, and 60 minutes. Compound concentrations were measured at these time-points using Liquid Chromatography with tandem mass spectrometry (LC-MS-MS). Pharmacokinetic studies were performed by orally dosing test compounds at 30 mg/kg dissolved in 50% PEG400 and 50% water in female athymic nude mice. Plasma and brain tissue concentration were measured using mass spectrometry (MS).

### siRNA studies

On Target-Plus Smart-pool siRNAs (Horizon Discovery) targeting key DNA repair and control genes were reconstituted as 1 μM stock solutions in 1X siRNA buffer made from 5X siRNA buffer (Thermo Fisher Scientific) (Supplementary Table 3). Deconvoluted siRNAs were reconstituted as 10 μM stock solutions in 1X siRNA buffer. The EJ-DR cells were seeded at 1000 cells/well in a 384-well plate and transfected with 10 pmol of Smart-pool siRNAs along with a non-targeting control. 72 hours after transfection, the DSB ligands were added along with mibefradil as a control. 24 hours after DSB ligand addition, the plates were rinsed and 72 hours later the plates were imaged and analyzed as described above.

### Quantitative reverse transcription PCR (RT-qPCR)

RNA samples were lysed in TRIzol (Invitrogen) and then reverse transcribed using the TaqMan RT-qPCR kit according to manufacturer’s instructions by ARQ Genetics. TaqMan fluorescent probes for mTOR and GAPDH were used and mRNA expression was quantified using ΔΔCt comparison normalized to the GAPDH control.

### Statistical analysis

Data are presented as the mean ± SEM and the analyses were performed using Microsoft Excel and GraphPad Prism Software. Statistical comparisons were conducted using Student’s two-tailed *t-test* and described significant as ^*^ *p* < 0.05, ^**^ *p* < 0.01, ^***^ *p* < 0.001.

## SUPPLEMENTARY MATERIALS



## Abbreviations

GBM: Glioblastoma
CNS: central nervous system
RT: radiation therapy
CCB: calcium channel blocker
DSB: double strand break
hERG: human Ether-à-go-go-Related Gene
CYPs: cytochrome P450 enzymes
TMZ: temozolomide
NHEJ: non-homologous end joining
HR: homologous recombination
cNHEJ: canonical non-homologous end joining
alt-NHEJ: alternative non-homologous end joining
DDR: DNA damage response
SI: selectivity index
SAR: structure activity relationship
C_max_: maximum concentration achieved
