# Supplementary material for: Creation of a new class of radiosensitizers for glioblastoma based on the mibefradil pharmacophore
Source: Oncotarget. 2021 Apr 27;12(9):891–906. doi: 10.18632/oncotarget.27933 (PMC8092340; doi:10.18632/oncotarget.27933)
Supplement: Supplementary file 1 [file oncotarget-12-891-s001.pdf]

Creation of a new class of radiosensitizers for glioblastoma based on the mibefradil pharmacophore

SUPPLEMENTARY MATERIALS

General

The reagents and solvents used for synthesis were of reagent grade quality. Dry solvents were purchased and used as such. All compounds were individually purified by chromatography on silica gel or by recrystallization and were of > 95% purity for characterization purposes as determined by LCMS using UV absorption at 220 or 280 nM and/or NMR integration. In practice compounds were not always purify to > 95% purity prior to using in the next synthetic step and often crude material was of sufficient purity and was carried forward.

The synthesis schema for the 13 analogues of mibefradil referred to in the main text are summarized below.

Synthesis of tetrahydronaphthalene derivatives I

YU241553, YU244210, YU252218, YU252222, YU252223, YU252373, YU252376, YU252377 were synthesized according to the following generalized scheme:

Scheme 1

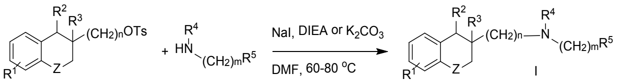

Preparation of tetrahydronaphthalene core intermediates

Intermediate: tert-butyl 2-[(1S,2S)-6-fluoro-2-hydroxy-1-isopropyl-tetralin-2-yl] acetate (2)

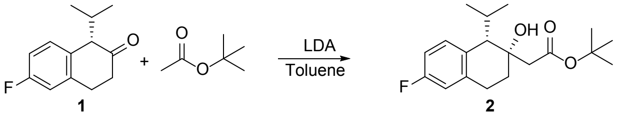

Step 1: A solution of LDA (1 M, 28.12 mL) in 2-methyl-THF was added to a cold (–78°C) stirred solution of tert-butyl acetate (3.40 g, 29.25 mmol) in toluene (16.00 mL) under nitrogen and the mixture was stirred at –78°C for 1 h.

Step 2: In a separate flask (1S)-6-fluoro-1-isopropyl-tetralin-2-one (2.32 g, 11.25 mmol) was dissolved in toluene (48.00 mL) and cooled to –78°C. Then t-butyl acetate lithium enolate solution prepared in step 1 (~44 mL, 28.12 mmol) was added to the above solution over 15 min under nitrogen and the mixture was stirred at –78°C for 3–4 h and then kept in a freezer at –20°C under nitrogen overnight. The reaction mixture was poured into an ice-cold 1N HCl (40 mL) solution and the product was extracted with ethyl acetate, washed with water, brine, dried (Na_2SO_4), concentrated to a viscous oil (4.37 g) and purified by Biotage MPLC (0–20% EtOAc-hexanes with gradient elution) to afford tert-butyl 2-[(1S,2S)-6-fluoro-2-hydroxy-1-isopropyl-tetralin-2-yl] acetate (3.52 g, 10.92 mmol, 97% yield) as a clear oil. ^{1}H NMR (400 MHz, Chloroform-*d*) δ 6.94 (tt, *J* = 6.8, 3.7 Hz, 1H), 6.84–6.76 (m, 2H), 4.15–4.08 (m, 1H), 2.99–2.90 (m, 1H), 2.81–2.68 (m, 1H), 2.56 (t, *J* = 2.4 Hz, 1H), 2.44 (pd, *J* = 7.0, 2.6 Hz, 1H), 2.33 (d, *J* = 15.6 Hz, 1H), 2.27 (dd, *J* = 15.6, 0.7 Hz, 1H), 2.16–2.06 (m, 1H), 1.75 (ddt, *J* = 13.8, 8.0, 1.9 Hz, 1H), 1.44 (s, 9H), 1.11 (d, *J* = 7.0 Hz, 3H), 0.44 (d, *J* = 6.9 Hz, 3H). LCMS: m/z 249.0 [M-OH-^{t}Bu]$^+$

Intermediate: (1S,2S)-6-fluoro-2-(2-hydroxyethyl)-1-isopropyl-tetralin-2-ol (3)

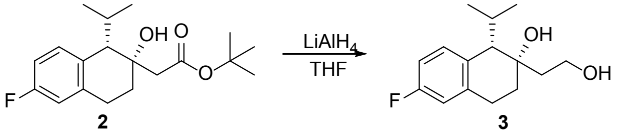

A solution of tert-butyl 2-[(1S,2S)-6-fluoro-2-hydroxy-1-isopropyl-tetralin-2-yl]acetate (3.52 g, 10.92 mmol) in THF (30 mL) was added to a stirred cold (0°C) suspension of $LiAlH_4$ (738 mg, 19.5 mmol) in THF (30 mL) and the mixture was stirred at 0°C for 2 h. Reaction was quenched by addition of water (0.74 mL), 15% NaOH (0.74 mL), water (2.2 mL) consecutively and the mixture was stirred at rt until excess LAH is fully quenched and white precipitate of $NaAl_2O_3$ is formed which was filtered off. The filtrate was dried over an. MgSO4, concentrated and purified by Biotage MPLC (EtOAc-hexanes, 12-100% gradient/10 CV) to afford (1S,2S)-6-fluoro-2-(2-hydroxyethyl)-1-isopropyl-tetralin-2-ol (2.32 g, 9.19 mmol, 84.20% yield) as a clear viscous oil. ^{1}H NMR (400 MHz, Chloroform-*d*) δ 6.99 (dd, *J* = 8.2, 5.9 Hz, 1H), 6.81 (tq, *J* = 9.7, 2.8, 2.3 Hz, 2H), 3.88 (ddd, *J* = 5.9, 4.9, 1.0 Hz, 2H), 3.02–2.91 (m, 1H), 2.83–2.73 (m, 1H), 2.65 (t, *J* = 2.3 Hz, 2H), 2.37 (heptd, *J* = 7.0, 2.5 Hz, 1H), 2.17–2.08 (m, 1H), 1.87 (ddt, *J* = 13.5, 8.2, 2.0 Hz, 1H), 1.67 (td, *J* = 5.9, 4.7 Hz, 2H), 1.15 (d, *J* = 6.9 Hz, 3H), 0.48 (d, *J* = 6.9 Hz, 3H). LCMS: m/z 235.1 [M-OH]$^+$

Intermediate: 2-[(1S,2S)-6-fluoro-2-hydroxy-1-isopropyl-tetralin-2-yl] ethyl 4-methylbenzenesulfonate (4)

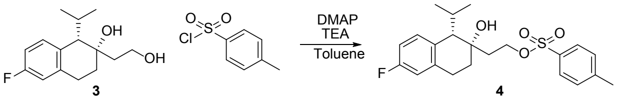

Neat 4-methylbenzenesulfonyl chloride (2.10 g, 11.03 mmol) was added in small portions to a cold (0–5°C) stirred solution of (1S,2S)-6-fluoro-2-(2-hydroxyethyl)-1-isopropyl-tetralin-2-ol (2.32 g, 9.19 mmol), TEA (1.12 g, 11.03 mmol, 1.53 mL) and DMAP (112 mg, 919.00 umol) in toluene (25 mL) and the mixture was stirred at rt overnight. The reaction was quenched with ice water and extracted with ether, washed with cold 1N HCl, water, brine, dried (MgSO4) and concentrated. Crude isolate was purified by Biotage MPLC (0–30% gradient elution with EtOAc-hexanes) to afford 2-[(1S,2S)-6-fluoro-2-hydroxy-1-isopropyl-tetralin-2-yl] ethyl 4-methylbenzenesulfonate as a viscous oil which solidified as an off-white solid upon standing. ^{1}H NMR (400 MHz, Chloroform-*d*) δ 7.79–7.74 (m, 2H), 7.37–7.32 (m, 2H), 6.91 (dd, *J* = 8.3, 5.9 Hz, 1H), 6.83–6.75 (m, 2H), 4.32–4.18 (m, 2H), 2.93 (dd, *J* = 18.3, 8.1 Hz, 1H), 2.80–2.67 (m, 1H), 2.47 (s, 3H), 2.46–2.44 (m, 2H), 2.26 (pd, *J* = 6.9, 2.6 Hz, 1H), 2.05–1.96 (m, 1H), 1.79 (dt, *J* = 15.0, 6.8 Hz, 1H), 1.74–1.63 (m, 2H), 1.10 (d, *J* = 6.9 Hz, 3H), 0.44 (d, *J* = 6.9 Hz, 3H). LC-MS: m/z 389.40 $[M-H_2O+H]^+$

Amination of tosylate intermediate (4): A schematic of the general procedure for the amination of tosylate intermediate 4 with amines HNR^1R^2

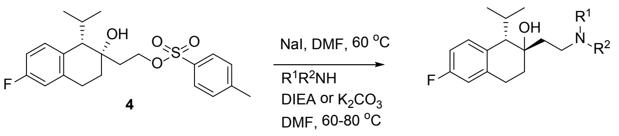

A stirred solution of tosylate (0.1 eq) and NaI (0.1 eq) in DMF (1 mL) was heated at 60°C for 1 h. Then corresponding amine (0.1 eq) and DIEA (0.1–0.3 eq) or K2CO3 (0.1–0.3 eq) were added and the mixture was heated at 60–80°C until amination is complete (typically 8–16 h depending on the reactivity of amine component). Reaction was to cool to rt and diluted with EtOAc, washed with satd. $NaHCO_3$, water, brine, dried (Na_2SO_4), concentrated and purified by silica gel FCC (gradient elution with 0–20% MeOH-DCM depending on the polarity of the product amines) to afford corresponding aminated products.

Synthesis of tetrahydronaphthalene derivatives II

The synthesis of YU241551 is described below.

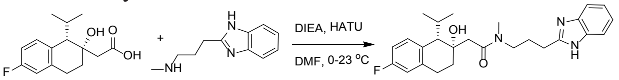

Neat HATU (Hexafluorophosphate Azabenzotriazole Tetramethyl Uronium) (76 mg, 201 umol) was added to a cold (0–5°C) solution of 2-[(1S,2S)-6-fluoro-2-hydroxy-1-isopropyl-tetralin-2-yl]acetic acid (54 mg, 201 umol), 3-(1H-benzimidazol-2-yl)-N-methyl-propan-1-amine (53 mg, 201 umol, bis HCl) and DIEA (130 mg, 1.0 mmol, 175 uL) in DMF (075 mL) and the mixture was stirred at rt for 3 h. Reaction was quenched with satd. NaHCO3, washed with water, brine, dried (Na2SO4), evaporated and purified by silica gel FCC (gradient elution with 0–10% MeOH-DCM over 10 CV) to afford N-[3-(1H-benzimidazol-2-yl)propyl]-2-[(1S,2S)-6-fluoro-2-hydroxy-1-isopropyl-tetralin-2-yl]-N-methyl-acetamide as a semi-solid. LCMS: 438.2 $[M+H]^+$

Synthesis of amide derivatives

Synthetic routes for the preparation of amide derivatives YU252374 and YU253106 (amide derivatives I) and YU252386 and YU252294 (amide derivatives II) is depicted below.

Synthesis of amide derivatives I

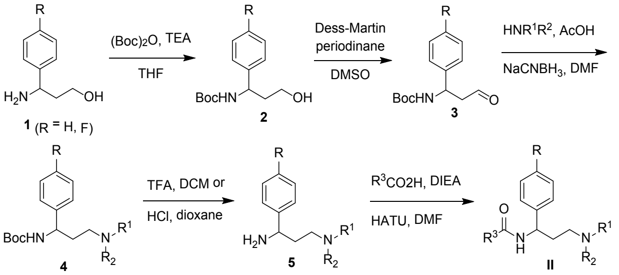

Generally, reaction of amine 1 with 1 equivalent of $(Boc)_2O$ and 10% TEA in THF under gentle reflux for ~1 h gave the corresponding Boc protected amine 2. The hydroxyl group of 2 was oxidized with Dess-Martin periodinane in DMSO to provide desired aldehyde 3. Reductive amination aldehyde 3 with secondary amines (R^1R^2NH) with $NaCNBH_3$ (1.5 equiv) in DMF afforded corresponding tertiary amines 4. Removal of Boc group of 4 followed by amide coupling of primary amine group with corresponding acids (R^3CO_2H) provided the desired amide derivatives, YU252374, YU253106.

Synthesis of amide derivatives II

The general procedure for preparation of the phenylacetamide derivatives YU252386 and YU252294, is outlined below.

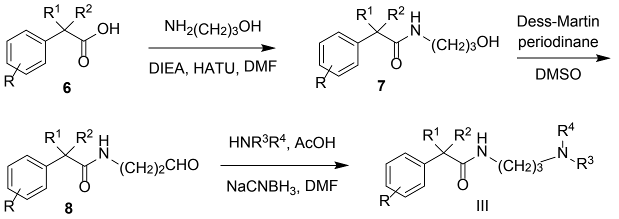

Amide coupling of acid 6 and 3-amino-1-propanol using HATU and TEA in DMF provided amides 7. The hydroxyl group of 7 was oxidized with Des-Martin periodinane in DMSO to give corresponding aldehydes 8. Reductive amination aldehyde 8 with secondary amines (R^3R^4NH) with $NaCNBH_3$ (1.5 equiv) in DMF afforded phenylacetamide derivatives YU252386 and YU252294.

Synthesis of YU252293

Synthesis of intermediate

1-chloro-4-((2-chloroethoxy)(phenyl)methyl) benzene

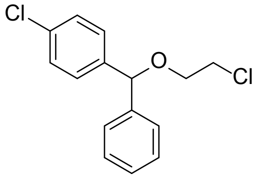

A neat stirred mixture of 1-chloro-4-(chloro(phenyl) methyl)benzene (1 g, 4.22 mmol) and 2-chloroethanol (1.70 g, 21.1 mmol) was heated at 130°C for 6 h. Excess 2-chloroethanol was evaporated under reduced pressure to afford 1-chloro-4-((2-chloroethoxy)(phenyl)methyl) benzene (1.15 g, 4.09 mmol, 97% yield) as a light brown oil. ^{1}H NMR (400 MHz, Chloroform-*d*) δ 7.36 (m, 4H), 7.32 (m, 5H), 5.43 (s, 1H), 3.77–3.72 (m, 2H), 3.72–3.68 (m, 2H). LCMS: m/z 281.1 $[M+H]^+$

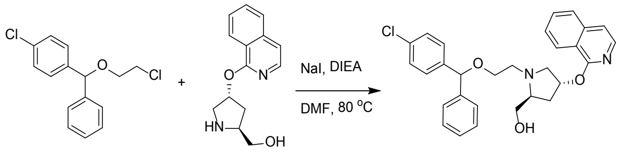

Synthesis of YU252293

A stirred solution of 1-chloro-4-[2-chloroethoxy(phenyl)methyl]benzene (30 mg, 104 umol), [(2S,4R)-4-(1-isoquinolyloxy)pyrrolidin-2-yl]methanol (17 mg, 53 umol, 2HCl), sodium iodide (8 mg, 52 umol) and DIEA (34 mg, 261 umol, 46 uL) in DMF (1 mL) was heated at 80°C for 12 h. Reaction was diluted with EtOAc, quenched with satd. NaHCO3, washed with water, brine, dried (Na2SO4), evaporated and purified by silica gel flash column chromatography (gradient elution with 0–20% MeOH-DCM over 10 CV) to afford [(2S,4R)-1-[2-[(4-chlorophenyl)-phenyl-methoxy]ethyl]-4-(1-isoquinolyloxy)pyrrolidin-2-yl]methanol (16.4 mg, 65%) as a mixture of diastereomers. LCMS: m/z 489.2 $[M+H]^+$.

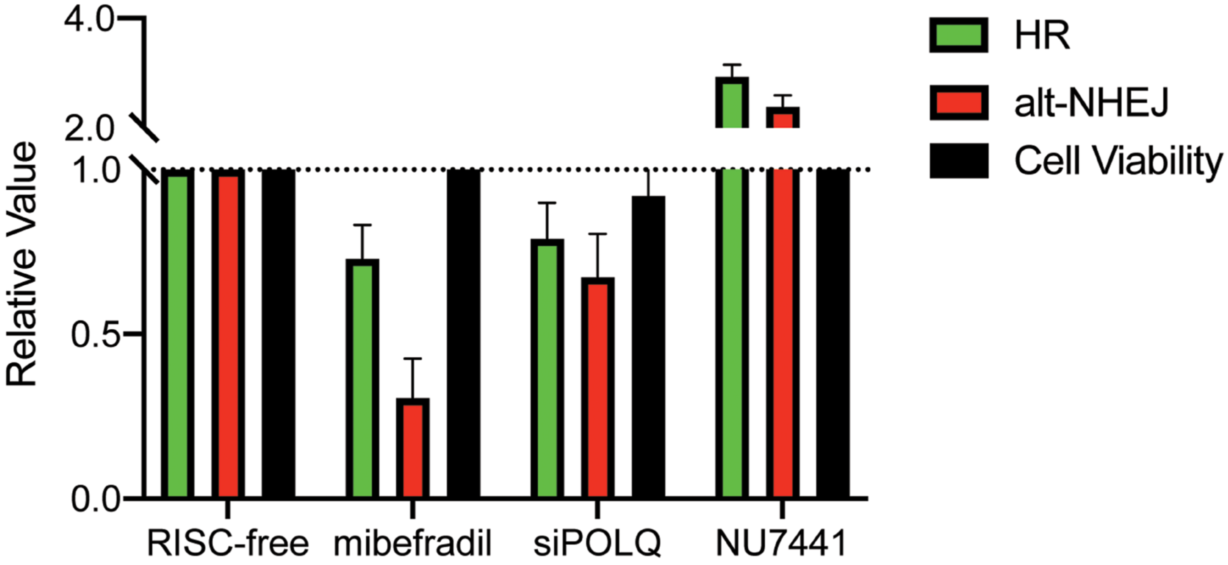

Supplementary Figure 1: Relative values of inhibition of HR, alt-NHEJ, and cell viability of controls RISC-free, mibefradil, NU 4771 (DNA-PK inhibitor) and a pooled siRNA targeting POLQ.

Supplementary Table 1: A summary of the pharmacokinetic (PK) parameters of mibefradil and three mibefradil analogues

Mean PK Parameter	Mibefradil	YU252222	YU252386	YU252377
Area under concentration vs time curve (AUC) (0-x) (ng*hr/mL)	4840	63.8	1350	175
AUC(0-x)/Dose (ng*hr/mL/mg/kg)	161	NA	45.0	5.83
AUC(0-∞) (ng*hr/mL)	4860	76.5	1460	177
AUC(0-∞)/Dose	162	NA	48.7	5.90
% AUC Extrapolation	0.4	16.6	7.5	1.1
Original Dose (mg/kg)	30	0	30	30
Maximum concentration (C_{max}) (ng/mL)	1090	11.3	338	68.9
C_{max}/Dose (ng/mL/mg/kg)	36.3	NA	11.3	2.30
Time at which C_{max} was observed (t_{max}) (hr)	1.0	2.0	1.0	1.0
Elimination half-life ($t_{1/2}$) (hr)	2.60	4.23	4.01	1.81
Regression Points (hr)	8, 12, 24	4, 8, 12	4, 8, 12	4, 8, 12

Compounds were orally dosed at 30 mg/kg in female athymic nude mice and parameters were estimated using Watson pharmacokinetic software 7.3.0.01 (Thermo Fisher Scientific) using a non-compartmental approach consistent with the route of administration. (NA = not applicable).

Supplementary Table 2: A summary of the alternative non-homologous end joining (alt-NHEJ), homologous recombination (HR), cytotoxicity (CT) IC50s and the selectivity index of multiple inhibitors in the DNA damage response

Target/Compound	alt-NHEJ IC50 (µM)	HR IC50 (µM)	CT IC50 (µM)	Selectivity Index (HR IC50/alt-NHEJ IC50)
**mTOR**				
AZD 8055	0.05 ± 0.01	2.85 ± 1.04	0.89 ± 0.28	57.00
Everolimus	0.05 ± 0.03	0.07 ± 0.03	3.17 ± 2.05	1.40
**S6K, CDK4/6, CHK1/2**				
PF-04708671 (S6K)	> 60	> 60	36.89 ± 3.82	-
Pablociclib (CDK4/6)	0.04 ± 0.01	0.06 ± 0.01	1.37 ± 0.36	1.50
AZD 7762 (CHK1/2)	> 60	> 60	0.13 ± 0.01	-
**AKT**				
MK-2206	0.66 ± 0.31	4.40 ± 5.17	16.48 ± 2.56	6.67
GDC-0068	9.40 ± 1.78	22.08 ± 1.84	41.49 ± 3.32	2.35
Afuresertib	0.61± 0.46	4.53 ± 5.75	14.24 ± 2.32	7.43
AZD 5363	16.91 ± 26.54	31.58 ± 59.23	48.45 ± 72.98	1.87

The IC50 values are the average of four technical replicates and are reported as mean ± SEM.

Supplementary Table 3: List of the 240 On Target-Plus Smart-pool siRNAs targeting key DNA repair genes and controls used in the siRNA studies

RISC-free + Mibefradil
siRPA2
siFRAP1
siUBE2A
siFEN1
siPOLA
siATRX
siCLK2
siTYMS
siTP73
siERCC6
siPOLE
siUVRAG
siTTRAP
siDNMT1
siSHFM1
siDLG7
siKUB3
siALKBH2
siPOLQ
siERCC2
siTNP1
siCHEK1
siCHEK2
siKIAA1018
siXPA
siPOLL
siGTF2H3
siGTF2H1
siGCN5L2
siYBX1
siEYA3
siCSNK1E
siXAB2
siTCEA1
siFANCD2
siUBE2V2
siRTEL1
siERCC4
siRENT1
siBAZ1B
siGTF2H2
siPOLD1
siPRPF19

siPOLS
siDDB1
siMSH2
siTP53
siADPRTL3
siUNG
siPOLH
siBRCA1
siERCC3
siMSH4
siTREX1
siBTG2
siHRMT1L6
siTADA3L
siPOLI
siRAD23B
siLIG3
siCETN2
siREV3L
siPMS2
siVCP
siTOP2A
siSOD1
siHMGB2
siMUS81
siCSNK1D
siMJD
siRAD18
siMGMT
siAPEX1
siC7ORF11
siAPTX
siEME2
siRAP80
siMUTYH
siATM
siC11ORF13
siCIB1
siNPM1
siBRIP1
siMSH3
siPARP2
siXPC
siRRM2
siRUVBL2
siGIYD1

siTDP1
siRAD51C
siRAD23A
siSETMAR
siMEN1
siHMGB1
siMMS19L
siHTATIP
siTRIP13
siDMC1
siEXO1
siMGC32020
siFANCG
siCHAF1A
siFANCF
siCXORF53
siPMS1
siUSP1
siNEIL2
siXRCC2
siIGHMBP2
siKIAA1596
siMSH5
siRAD51L1
siTRIM28
siPNKP
siERCC5
siRBBP8
siRNF8
siSIRT1
siBLM
siFANCC
siPOLB
siABL1
siFANCA
siCNOT7
siRPS27L
siFANCB
siATF2
siRAD54B
siDCLRE1C
siCCNH
siFLJ40869
siRAD51
siFLJ12610
siPOLM

siRAD21
siMDC1
siALKBH
siLIG4
siFANCE
siNEIL3
siRECQL5
siCSPG6
siRRM2B
siPOLG2
siMLH1
siUNG2
siPOLK
siFANCL
siUBE2B
siRAD50
siPARP1
siATRIP
siRPA1
siPER1
siRNF168
siIHPK3
siRAD17
siHEL308
siNTHL1
siDCLRE1A
siNEIL1
siRAD9A
siRECQL
siMNAT1
siHUS1
siPARG
siMGC4189
siGADD45A
siCDKN2D
siPOLE2
siMSH6
siPCNA
siSPO11
siWRN
siDDB2
siERCC1
siPOLG
siGTF2H5
siTP53BP1
siREV1L

siNUDT1
siCKN1
siRPA3
siPMS2L5
siFLJ10719
siMGC2731
siCDK7
siBRCA2
siSMC1L1
siLIG1
siPRKCG
siGADD45G
siRAD52B
siDDX11
siC2ORF13
siFLJ21816
siDUT
siUBE2V1
siPOLN
siSMC6L1
siMPG
siTOPBP1
siXRCC1
siDCLRE1B
siNBS1
siMAD2L2
siHSU24186
siRAD54L
siRAD1
siDEPC-1
siTDG
siMRE11A
siTREX2
siOGG1
siATR
siAPEX2
siH2AFX
siRECQL4
siBRE
siMBD4
siFLJ13614
siKIAA0625
siRAD51L3
siSMUG1
siGTF2H4
siXRCC3

siG22P1
siPRKDC
siEYA1
siDNA2L
siFLJ22833
siPLK1
siRAD52
siEME1
siMIZF
siMLH3
siXRCC5
siXRCC4
siPRKDC #8
RISC-free + NU7441
siUBE2N
siASF1A
